# Inappropriate Protocol of Implant Placement in Contact with Impacted Teeth Leading to Failure

**DOI:** 10.1155/2023/7328891

**Published:** 2023-06-20

**Authors:** Imed Ouni, Lamia Mansour

**Affiliations:** ^1^Prosthetic Department, Dental Clinic of Monastir, University of Monastir, Monastir, Tunisia; ^2^ABCDF Laboratory for Biological, Clinical and Dento-Facial Approach, University of Monastir, Avicenna Avenue, Monastir 5019, Tunisia

## Abstract

Tooth impaction is a frequent phenomenon in patients with craniofacial syndrome, and the oral rehabilitation of such cases is considered a therapeutic challenge for the clinician. Placing implants in contact with impacted teeth may provide an alternative treatment for patients who refuse invasive surgery, and for whom orthodontic traction and surgery are not possible. However, the absence of evidence-based guideline protocols may sometimes lead to inappropriate execution by the clinician. This study aims to describe a case of early failure of an implant placed in contact with dental tissue and to identify the factors associated with implant failure to uncover and prevent their causative mechanisms.

## 1. Introduction

Tooth impaction is a frequent phenomenon in patients with craniofacial syndrome, as reported in several studies [1–3] that strongly influences the normal development of the craniofacial complex, which leads to aesthetic and functional disabilities that sometimes require an unconventional approach [4, 5]. Implant rehabilitation of the edentulous site can be solved by invasive surgery to extract impacted teeth followed by bone regeneration and implant placement or by anchoring the implant directly through the impacted teeth to avoid complex clinical procedures leading to big bone damage [6], especially when impaction is asymptomatic and multiple.

Transdental implants, or implants placed in contact with dental tissue, were tried by some authors [7–9] and showed good results in terms of stability and post-loading efficiency. Labidi et al. [10], in a literature review, reported 32 implants placed through impacted teeth with follow-up periods ranging from 6 months to 8 years. All the implants were clinically and radiologically stable except for one case of failure after 4 months of placement and one implant that presented bone loss on the mesial wall.

However, the generalization of this unconventional technique in daily practice requires an evidence-based guideline protocol, especially for cases with syndromic dental impaction, to ensure long-term stability and efficiency.

This study describes a case with multiple dental impactions associated with the dental syndrome who was rehabilitated by implants anchored in dental tissue. The lack of clear recommendations about the protocol led to inappropriate execution by the clinician.

## 2. Case Report

A 22-year-old female patient with amelogenesis imperfecta (AI) presented with the main complaint of compromised chewing function due to missing teeth in the third quadrant.

The patient stated that she was treated with heat-cured temporary crowns on the remaining teeth as well as an acrylic removable partial denture (RPD) in the mandibular edentulous ridge. However, she found chewing difficulties that led her to remove her RPD just one week later. She claimed denture displacement at each opening and closing movement associated with lesions of the oral mucosa.

**Figure 1:**
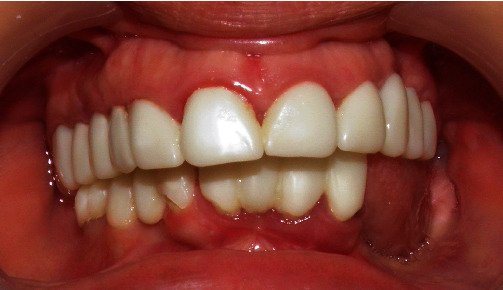
Intra-oral image showing full coverage provisional restorations with poor oral hygiene, mandibular partial edentulism with knife-edge residual ridge.

Oral examination showed a unilateral partially edentulous mandibular arch (Kennedy class II) with a knife-edge residual ridge, a lack of keratinized gingiva, and generalized gum inflammation. Provisional crowns were made with adequate vertical dimensions of occlusion but with a compromised aesthetic result (Figure 1).

**Figure 2:**
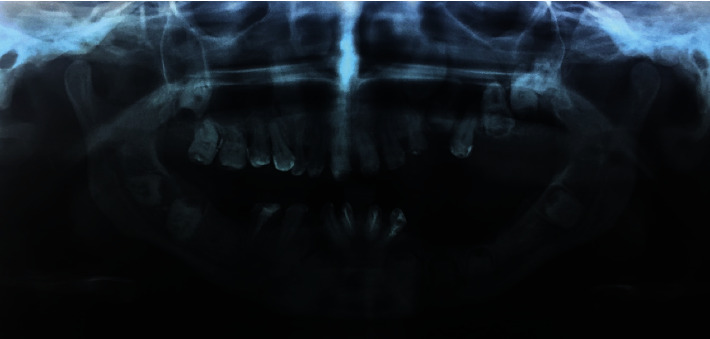
Panoramic radiograph showing deeply impacted multiple teeth and severe alveolar ridge deficiency in the left lower mandibular arch.

As shown in Figure 2, radiographs revealed the presence of multiple deeply impacted teeth and a severe alveolar ridge deficiency in the left lower mandibular arch.

Three options for rehabilitating mandibular edentulism were discussed with the patient. She refused all forms of removable devices, as well as invasive surgery to remove impacted teeth. Therefore, it was suggested to place the implants in contact with the impacted teeth.

The patient was informed about the treatment protocol as well as the possible complications and risks, and written consent was obtained.

**Figure 3:**
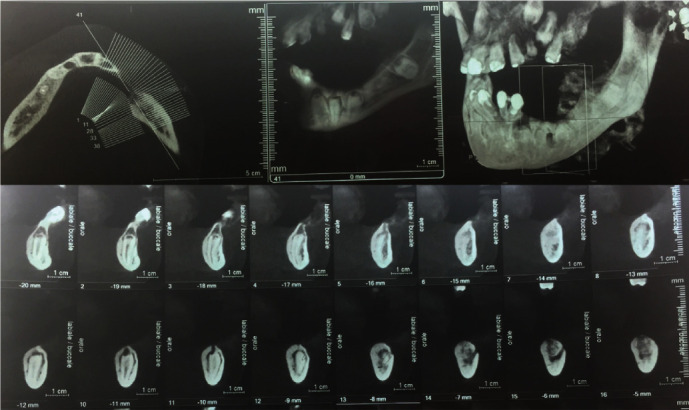
Cone-beam CT scan showing a small amount of bone only in the interdental spaces.

A cone-beam computed tomography (CT) scan showed that there was only a small amount of bone in the interdental spaces. Two implants (Easy System Implant, Chavanod, France), 3.7 mm in diameter, were placed using a submerged surgical procedure. One implant (11.5 mm in length) was placed between the impacted canine and the first premolar, and the other one (10 mm in length) was placed between the first and second premolars because of the proximity of the alveolar nerve and the low quantity of bone posteriorly, as shown in Figure 3. The implants were inserted following the complete drilling sequence with abundant irrigation due to the rigidity of the dental tissues.

**Figure 4:**
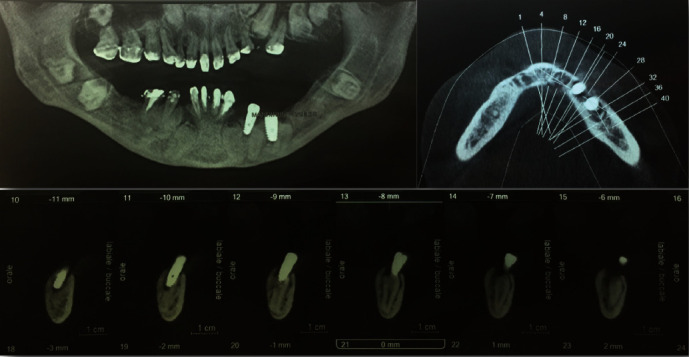
Cone-beam CT scan showing an exfoliation and bone loss around the implant placed between the impacted canine and the first premolar. The second implant placed between the first and second premolars is integrated into the bone and dental tissue in contact.

The healing process after surgery was uneventful. One month later, we noticed gingival dehiscence with exposed threads of the first implant (implant placed in the canine position) and slight mobility on contact with no pain or swelling. A cone-beam CT scan showed bone loss around the implant with no reduction in peri-implant bone height; it was like the implant had exfoliated from its socket. The second implant, however, was well integrated with the bone and dental tissue (Figure 4).

**Figure 5:**
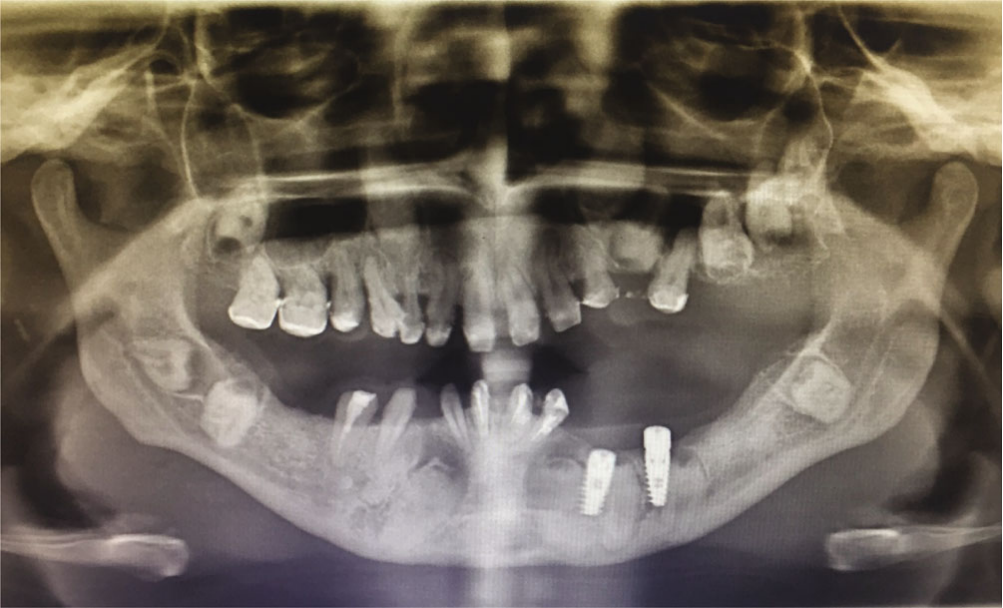
Panoramic radiograph showing wider fixture placed deep through the impacted canine and first premolar.

The failed fixture was removed, the surgical site was rinsed with chlorhexidine 0.12%, and an immediately wider-bodied fixture (4 mm in diameter and 11.5 mm in length) was placed deep through the impacted teeth following the same osteotomy site (Figure 5).

Rigorous follow-up visits were scheduled throughout the healing phase, and a periapical radiograph was taken at each visit to check radiolucent images at the tooth-to-implant or bone-to-implant interfaces.

**Figure 6:**
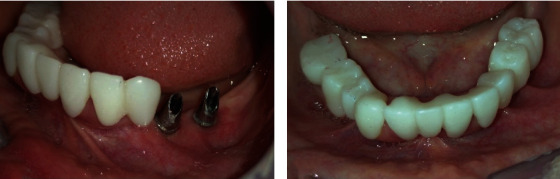
(a) Intra-oral image showing good gingival health around the abutment. (b) Occlusal view of full arch zirconia crowns.

Two months after surgery, both implants anchored in the unerupted teeth healed normally. Healing abutments were connected, and twenty days later, zirconia crowns were performed in association with a custom screw-retained abutment to improve the emergence profile of the crowns (Figure 6).

**Figure 7:**
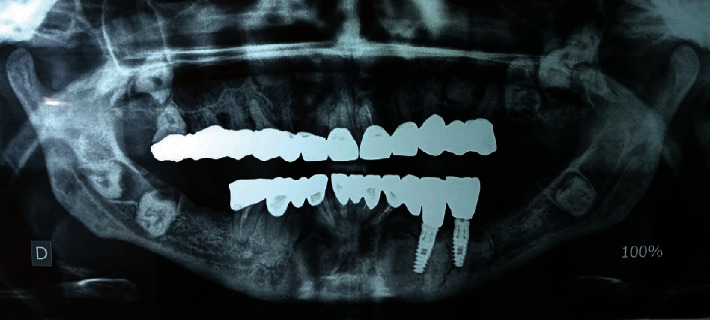
Panoramic radiograph after 48 months of follow-up. No changes were noted around implants, impacted teeth, or bone levels.

The patient was recalled for a check-up every six months, undergoing the radiographic and clinical assessment established by Cochran et al. [11], and to date (up to 36 months of follow-up) no symptoms or signs of prosthetic or implant failure have been noticed (Figure 7).

The patient expressed satisfaction with the masticatory efficiency and was grateful to have avoided the invasive surgical extraction of impacted teeth.

## 3. Discussion

Multiple dental impactions are often associated with syndromes, such as Gardner's syndrome, AI, or cleidocranial dysplasia [3, 12]. Oral rehabilitation in such cases is considered a therapeutic challenge for the clinician.

The “gold standard” treatment is the extraction of impacted teeth with bone regeneration and immediate implant placement [13]. However, patients often refuse this approach, which is considered more invasive and increases treatment cost and duration, particularly when the residual bone does not exhibit a sufficient thickness, as in our case, making simultaneous implant placement difficult.

For this reason, anchoring the implant in the impacted teeth is an alternative strategy more acceptable to the patient. This approach attempts to simplify the surgical phase, shorten treatment time, and improve esthetic outcomes since the impacted crowns provide better support for soft tissues [14].

In a case series [7, 9], Davarpanah et al. reported no adverse events at impacted tooth-to-implant interfaces with successful mid- to long-term survival.

Nonetheless, this case revealed some fundamental facts; the failure of the first implant to integrate may call into question the lack of an evidence-based consensus guideline for implant placement through impacted teeth. Several related questions come to mind: What is the minimum bone quantity necessary to ensure primary stability? Can we place the entire implant in contact with the dental tissue? Which dental tissue should be in contact with the implant? What is the minimum duration for healing?

In the absence of such a guideline, the placement of implants relies primarily on empirical experience. The causes of the failure of the first implant will be evaluated based on two possible causes:

(1) The implant was put in contact only with the enamel of the impacted tooth; however, histological data reported a possible mineral reaction only if the implant surface had an interface with dentin or cement [15, 16]. These findings agree with those reported by Davarpanah and Szmukler-Moncler, in which a short implant placed in contact with the enamel of an impacted canine has been lost. The authors suggested the use of a longer implant to pass through the dentin and pulp chamber [8].

(2) The implant was not surrounded by enough bone, especially in the cervical and apical parts. This is why, when it was placed deeper, we found better osteointegration.

Postsurgical radiographic controls are not very detailed for cases treated with this unusual technique. It is difficult to differentiate any detail on periapical or panoramic radiographs because of the superimposition of the radiopaque implants on the dental tissue of the impacted teeth. In three-dimensional cone-beam CT scans, the big difference in radiodensity between the implant and surrounding dental tissue leads to radiographic burnout and difficulty reading details in the implant interfaces [17].

Equally important, there is no guideline for craniofacial syndromes with multiple dental impactions regarding the quantity and quality of remaining bones and the management of surrounding soft tissue.

How wise would it be to place an implant in close proximity to an impacted tooth? This is a question we need to ask ourselves when treating cases of multiple teeth impaction.

Before practicing any particular technique, the clinician should know the protocols, costs, and benefits, and at the same time be able to treat any arising complications in the future.

## 4. Conclusion

Before attempting implant placement in contact with impacted teeth, practitioners should have adequate expertise and experience. Unless clinicians develop sufficient competency in the procedure, results reported by experienced clinicians may not be reproduced. It is proposed that an evidence-based guideline be established for case selection and carrying on of the protocol, which will enable operators to make the correct decisions about opting for this unconventional approach, especially for syndromic patients with multiple tooth impaction. It is also proposed that a long-term follow-up cohort study be undergone to assess the merits of this technique compared with conventional implant placement.

## Data Availability

Data supporting this case report are available from the corresponding author or first author upon reasonable request.
